# Interleukin-6 in Natural and Pathophysiological Kidney Aging

**DOI:** 10.3390/cells15030225

**Published:** 2026-01-24

**Authors:** Kerim Mutig, Prim B. Singh, Svetlana Lebedeva

**Affiliations:** 1 Department of Biosciences, School of Medicine, Nazarbayev University, Astana 010000, Kazakhstan; prim.singh@nu.edu.kz; 2 Scientific Center of Genetics and Life Sciences, Sirius University of Science and Technology, 354340 Sirius, Russia; 3 Department of Human Anatomy and Histology, Sechenov First Moscow State Medical University (Sechenov University), 119991 Moscow, Russia; 4 Department of Pharmacology, Institute of Pharmacy, Sechenov First Moscow State Medical University (Sechenov University), 119991 Moscow, Russia

**Keywords:** kidney, aging, inflammaging, inflammation, interleukin-6, cytokines, interleukin-6 inhibitor

## Abstract

Kidney aging is receiving growing attention in middle- to high-income societies due to increasing longevity in general population. Chronic Kidney Disease (CKD) has been widely accepted as a major non-communicable human disease affecting over 10% of the adult population in industrialized countries. CKD is mainly caused by metabolic and cardiovascular disorders such as diabetes mellitus and hypertension, disproportionally affecting older people, whereas natural kidney aging is driven by age-dependent systemic and renal low-grade inflammation. Interleukin-6 (IL-6) is the key cytokine mediating age-related inflammation. At the same time, IL-6 has been implicated in the pathophysiology of cardiovascular and renal disorders as a major pro-inflammatory cytokine. Thereby, IL-6 is placed at the intersection between natural and pathophysiological kidney aging, and the latter accelerates systemic aging and substantially limits life quality and expectancy. Growing clinical availability of IL-6 inhibitors for treatment of autoimmune and autoinflammatory disorders demands clarification of potential renal consequences as well. Available data suggests that IL-6 inhibition may be renoprotective in some kidney disorders, but the setting of kidney aging has received only minor attention. The present review focuses on the known effects of IL-6 associated with natural or pathophysiological renal aging.

## 1. Premise

Increasing life expectancy in the middle- to high-income societies is accompanied by the growing prevalence of renal insufficiency provoked either by natural kidney aging or metabolic and cardiovascular diseases disproportionally affecting older adults [1,2,3,4,5]. Natural kidney aging is characterized by gradual nephron loss during adult life leading to a slow decline in the glomerular filtration rate (GFR) by approximately 1 mL/min/1.73 m^2^ per year evident from the third decade of life on [6,7]. In the absence of primary kidney diseases and secondary kidney damage due to comorbidities, natural kidney aging shows a benign character and is not associated with disproportional increases in age-standardized risks of mortality or end-stage renal disease (ESRD) [7]. However, the aged kidney is prone to injury due to the limited compensatory ability and regenerative potential [8]. In fact, aging has been recognized as a significant and independent risk factor for both acute kidney injury (AKI) and chronic kidney disease (CKD) [2,9,10,11,12,13]. AKI is associated with high mortality and elevated risk of transition to CKD in older individuals [14]. Sustained deterioration of renal performance in advanced CKD constitutes a substantial challenge for the body’s homeostasis, provoking and aggravating multi-organ pathologies [15]. Older adults represent a large proportion of hemodialysis patients and patients with untreated kidney failure [16,17]. CKD, hemodialysis, and the associated comorbidities constitute a substantial socioeconomic burden rising with increasing longevity and a growing proportion of older adults in the general population [1,18]. Improved understanding of molecular (patho)physiology driving kidney aging is mandatory for strategies preserving renal function in advanced age.

## 2. Effects of Aging on Kidney Morphology and Function

Age-related deterioration of renal function is evident in healthy adults by a gradual decrease in GFR by roughly 1 mL/min/1.73 m^2^ per year beginning with the third decade of life [19,20]. This functional decline is caused by ongoing nephron loss throughout the adult life as mirrored by a measurable decrease in the parenchymal volume of the renal cortex from the age of 50 onwards [21,22]. Histologically, kidney biopsy specimens even from relatively healthy older adults such as living kidney transplant donors display a substantial loss of nephrons (up to ~50%) compared to younger counterparts [22]. The histological features of the aged kidney comprise glomerulosclerosis, tubular atrophy, tubulointerstitial fibrosis, and arteriosclerosis. In the absence of an underlying disease, the age-related alterations of renal morphology are commonly defined as senile or benign nephrosclerosis [23]. Benign nephrosclerosis itself bears no enhanced risk of ESRD but makes the aged kidneys prone to secondary damage in the case of the new onset of cardiovascular or metabolic diseases [2,23,24,25].

Healthy kidneys are capable of increasing GFR in response to physiological demands for stronger plasma filtration such as pregnancy, high dietary protein intake, or intensive physical activity [26,27,28]. The delta between the baseline and stimulated GFR values is referred to as the renal functional reserve (RFR) and reflects the compensatory capacity of the kidneys [29]. Apart from the physiological challenge, RFR is readily recruited in response to an acute nephron shortage as seen in the remaining kidney after unilateral nephrectomy [30,31]. Maladaptive activation of RFR occurs in diabetes mellitus leading to sustained hyperfiltration, glomerular hypertension, and ensuing kidney damage [32]. A gradual nephron loss in CKD is typically accompanied by augmented single nephron GFR (SNGFR) in remaining nephrons reflecting either the pathophysiological or compensatory hyperfiltration [33]. Consequently, RFR declines during CKD progression (Table 1) [34].

The gradual reduction of the nephron number during natural kidney aging does not lead to a compensatory increase of SNGFR in the remaining nephrons unless the nephron loss exceeds that expected for the age [7,38]. However, the critical deficit of nephrons in advanced age is likely compensated by hyperfiltration of the remaining nephrons as reflected by enlarged glomeruli in kidney biopsies from individuals over 70 years of age and suggested by SNGFR calculations in younger vs. older living kidney donors [7,38]. Furthermore, unilateral nephrectomy in older adults leads to the adaptive hyperfiltration of the remaining kidney similar to that observed in younger individuals [39]. Therefore, the aged kidney retains RFR which is activated either by an acute nephron deficit or at a critical stage of chronic nephron loss (Table 1). Apart from these conditions, natural kidney aging is accompanied by benign nephrosclerosis with a slow decline in GFR rather than compensation by RFR [7,38]. The age-related decline in renal function may reflect the general decrease of the body mass, metabolism, and physical activity with the respective reduction of the excretory demand. Altered renal hemodynamics may further limit the RFR response in aging [40].

Apart from the reduced GFR, aged kidneys display significant alterations of tubular functions making older adults prone to disorders of water and electrolyte homeostasis such as imbalances of sodium, potassium, or divalent cation homeostasis, metabolic acidosis, and underhydration [41,42].

Other aspects of the aging kidney are related to dysregulation of renal endocrine functions. A subset of peritubular fibroblast within the renal cortex produces erythropoietin (EPO), which is the key hormone driving erythropoiesis. The Baltimore Longitudinal Study on Aging revealed moderately elevated circulating EPO levels in relatively healthy older adults likely reflecting a compensatory response to the enhanced erythrocyte turnover [43]. Compensatory increases of EPO levels were further documented in geriatric patients with nutritional anemia [44]. While this data shows that natural kidney aging preserves a sufficient degree of EPO synthesis, advanced CKD has been strongly associated with increased risk of anemia in older adults [43,45,46]. Kidneys are also the main source of calcitriol, the active form of vitamin D, which is produced in proximal tubular cells by enzymatic processing of its precursor, 25-hydroxycholecalciferol. Lower serum calcitriol concentrations were associated with renal functional decline in older individuals which may reflect disfunction or damage of renal tubules [47]. Reduced renal performance, in turn, is an established risk factor for osteoporosis and fractures in aged individuals [48].

Taken together, natural kidney aging is associated with benign nephrosclerosis and gradual loss of nephrons underlying a slow decline in GFR after the third decade of life. The adaptive and regenerative potential of the aged kidney is limited, thus predisposing older adults to AKI or CKD. Cardiovascular and metabolic diseases disproportionally affecting older adults such as hypertension or diabetes accelerate the structural kidney damage leading to CKD.

## 3. Cell-Biological and Molecular Mechanisms of Kidney Aging

**Figure 1:**
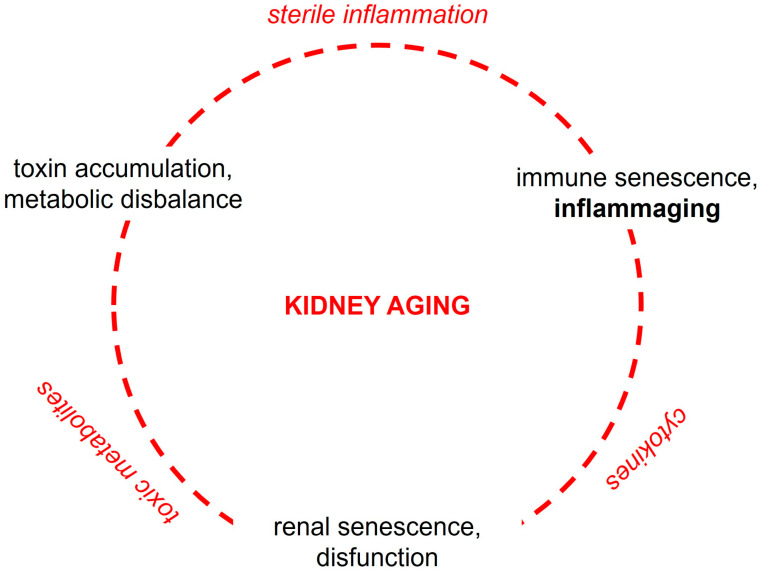
Vicious circle of pathophysiological events driving renal and systemic aging.

Renal aging is a complex and multifaceted cell-biological process driven by systemic and local mechanisms including genomic instability, cellular senescence, metabolic alterations, endocrine dysregulation, and inflammation [49]. The role of inflammation in the age-related organ disfunction has been increasingly recognized in recent decades leading to the concept of sustained low-level systemic inflammation accompanying natural aging introduced by Prof. Claudio Franceschi in 2000 and referred to as inflammaging [50,51]. Inflammaging describes alterations in the immune system during natural aging leading to enhanced production of pro-inflammatory cytokines and mediators [51]. The age-related changes in the immune system are driven by detrimental structural alterations in the primary and secondary immune organs, depression of innate and adaptive immunity, and compromised protective immune responses to exogenous or endogenous stressors [52]. At the same time, physical barriers such as skin and gut become leakier, thereby challenging the immune system by penetrating pathogens. Age-related suppression in the naive T-cells’ number and repertoire diversity with reduced presence of the critical immune receptors such as Toll-like and NOD-like receptors hamper the recognition and clearance of pathogens. Metabolic, cardiovascular, and other disorders disproportionally affecting older adults produce multiple internal stressors creating another challenge for the compromised immune system [53]. All these factors promote senescence of immune cells with acquisition of the senescence-associated secretory phenotype (SASP) characterized by pro-inflammatory cytokines and mediators including the interleukin-6 (IL-6). Persistent overexposure of the kidney tissue to circulating pro-inflammatory factors associated with inflammaging compromises the intact functionality of highly specialized kidney cell types provoking their senescence as well [2]. Accumulation of senescent cells in the aged kidney tissue is associated with enhanced local production of inflammatory cytokines [54,55]. In the context of kidney aging, cellular senescence describes the transition of differentiated kidney cells to a state of permanent cell cycle arrest with acquisition of SASP comprising cytokines, chemokines, growth factors and proteases exerting autocrine, paracrine, or endocrine effects [56]. The SASP acquisition is driven by impaired interactions between cellular organelles leading to a chain of pathophysiological events including mitochondrial dysfunction, inadequate energy metabolism, dysregulation of cellular proteostasis, endoplasmic reticulum (ER) stress, enhanced intracellular calcium release, and accumulation of reactive oxygen species (ROS) [57]. Apart from natural kidney aging, senescence has been implicated in the pathophysiology of acute and chronic kidney diseases [56]. AKI has been shown to provoke cellular senescence via immunologic and epigenetic mechanisms triggered by hypoxia and reactive oxygen species (ROS) generation [58]. Senescence limits the regenerative capacity of glomerular, tubular, and interstitial kidney cells during AKI resolution and increases the risk of an ensuing transition to CKD [59]. CKD has been associated with the accumulation of senescent cells in the renal tissue and global immunosenescence promoting premature kidney and systemic aging (Figure 1) [59]. Renal senescence leads to downregulation of the anti-aging *KL* gene with detrimental implications for health and lifespan [59,60]. The *KL* gene is abundantly expressed in proximal and distal tubular epithelia and encodes the a-Klotho (Klotho), which is a single-pass transmembrane protein serving as a co-receptor for the fibroblast growth factor-23 (FGF23) and thereby participating in the regulation of phosphate and calcium homeostasis [61]. Apart from the membrane-resident Klotho, its soluble forms produced either by proteolytic cleavage or alternative splicing are secreted into blood and urine to interfere with several molecular pathways including the mammalian target of rapamycin (mTOR), wingless-type MMTV integration site (Wnt), and transforming growth factor beta (TGF-b) signaling pathways [61,62,63]. Both membrane-bound and soluble Klotho variants retard renal and systemic aging. In a simplified view, the membrane-bound Klotho promotes renal phosphate excretion, thus preventing hyperphosphatemia and vascular calcification, whereas the soluble Klotho exerts complex antioxidant, anti-inflammatory, and antifibrotic effects [60,61,62,63]. In contrast, an inflammatory environment suppresses the Klotho expression, thus accelerating renal aging and susceptibility to disease [64]. SASP of kidney cells combines cytokines, chemokines, growth factors, and proteases creating the pro-inflammatory microenvironment. Several of these factors such as the tumor necrosis factor (TNF), TGF-β, interleukin 1β (IL-1b), and IL-6 have been strongly associated with CKD [65]. IL-6 has received major attention in gerontology since this cytokine appears to serve as a bridge between natural aging and age-associated morbidity [66,67].

## 4. Principles of Interleukin-6 Signaling

Interleukin-6 (IL-6) belongs to the four-helical cytokine family and is a glycoprotein with molecular weight of approximately 26 kDa produced by T and B lymphocytes, monocytes, fibroblasts, keratinocytes, mesangial cells, endothelial cells, and subsets of epithelial cells [68]. IL-6 is a pleiotropic cytokine with a variety of cell-biological and physiological effects related to immunomodulation and metabolism [69,70,71,72]. The IL-6 signaling requires the glycoprotein 80 (gp80) serving as the cognate a-subunit of the IL-6 receptor (IL-6R) and gp130 serving as the b-subunit shared by all IL-6 family cytokines. IL-6R (gp80) is expressed in certain types of immune cells, hormone-secreting hypothalamic or pituitary cells, and epithelial cells, whereas gp130 exhibits a ubiquitous expression pattern. Binding of IL-6 to the membrane-resident IL-6 receptor (mIL-6R) elicits the classical signaling mode which mediates adaptive and cytoprotective effects in physiological settings [70,71,73,74,75]. While the classical IL-6 signaling takes place in cell types expressing IL-6R, broader action of the cytokine is enabled by the trans-signaling relying on the soluble receptor form (sIL-6R) released by neutrophils, monocytes, T helper cells, and hepatocytes via proteolytic cleavage of mIL-6R [76,77]. The binding of IL-6 to sIL-6R in the extracellular fluid builds a preformed complex interacting with widely expressed gp130 and initiating the IL-6 signaling in a broad cell-type spectrum independently on the presence of mIL-6R. The trans-signaling is believed to mediate the inflammation-related effects [76]. Apart from the classic and trans-signaling modes, presence of the IL-6:IL-6R complex on the membrane of one cell may elicit signaling in a gp130-expressing neighboring cell via a close spatial interaction permitting formation of the joint IL-6 signaling complex. This signaling mode has been termed trans-presentation or cluster signaling and may take place between antigen-presenting dendritic cells transmitting the IL-6:IL-6R complex to Cluster of Differentiation 4 (CD4)-expressing T-cells to promote their differentiation towards T helper 17 (Th17) cells [78]. This juxtacrine signaling mode has been implicated in neuroinflammation and autoimmunity by experimental studies [79]. The balance between the adaptive and inflammatory effects of IL-6 is maintained by the soluble gp130 form (sgp130) produced by alternative splicing or proteolytic cleavage [80,81,82]. By absorbing the circulating IL-6:sIL-R6 complexes, sgp130 serves as an endogenous antagonist for the trans-signaling and trans-presentation while preserving the classical signaling mode [76,83]. However, sgp130 does not affect the autocrine IL-6 signaling which takes place in certain types of immune and non-immune cells owing to concomitant expression of IL-6, IL-6R, and gp130 enabling intracellular formation of the IL-6 signaling complex [83,84,85,86]. All IL-6 signaling modes share the downstream signal transducing pathway mediated by Janus Kinases (JAK) providing activating phosphorylation to the Signal Transducer and Activator of Transcription 3 (STAT3) with ensuing expression of genes related to cell proliferation, survival, or inflammation depending on the context [68]. STAT3 represents the common molecular pathway integrating effects of distinct cytokines with pro- and anti-inflammatory effects in part via the downstream Suppressors of Cytokine Signaling (SOCs) acting as a negative feedback for the cytokine-STAT3 signaling [87].

## 5. Systemic Interleukin-6 Signaling During Aging

High variability of circulating IL-6 levels precludes a clear definition of its normal range in healthy adults. A recent meta-analysis of available data on IL-6 plasma levels in healthy adults provided a pooled mean value of approximately 5.19 pg/mL, whereas the individual values varied from 0 to 43.5 pg/mL [88]. The same meta-analysis revealed a gradual increase in circulating IL-6 levels with age by about 0.05 pg/mL per year [88]. Effects of aging on the soluble IL-6R and gp130 variants have not been systematically evaluated. A study focusing on IL-6 signaling in the postmenopausal period of life revealed gradual, age-associated increases of the serum sIL-6R and sgp130 levels in women until the seventh decade with ensuing reduction in older ages for both proteins [89]. In that study, serum levels of IL-6, sIL-6R, and sgp130 were significantly higher during the first 10 years of menopause and the increase in serum sIL-6R levels was negatively associated with bone mineral density suggesting an association of the IL-6 trans-signaling with postmenopausal risk of osteoporosis [89]. Significantly enhanced plasma IL-6 and sgp130 levels along with a strong trend towards increased sIL-6R levels have been also reported in older adults with insulin resistance and hypertriglyceridemia suggesting pathophysiological effects of the trans-signaling in diabetes mellitus and metabolic syndrome disproportionally affecting older adults [90]. Despite the well-recognized pathophysiological role of the trans-signaling in different disorders including kidney diseases, enhanced sIL-6R serum levels have been associated with a lower risk of dementia in older women suggesting potential neuroprotective effects of the trans-signaling [91]. Interestingly, the IL-6 trans-signaling mode has been shown to mediate not only pathophysiological but also renoprotective effects in distinct AKI mouse models [92,93].

Plasma IL-6 levels follow a biphasic circadian pattern in healthy young adults, but this rhythm is flattened in older adults [94]. Thus, advanced aging is associated with enhanced plasma IL-6 levels and an altered circadian pattern of IL-6 secretion. Chronically elevated plasma IL-6 levels may contribute to desynchronized activity of the hypothalamic-pituitary adrenal (HPA) axis and sleep/awake rhythm frequently reported in aged adults [88,94,95,96,97]. IL-6 has been increasingly recognized as the central player in inflammaging as its levels strongly correlate with age-related diseases, disability, and mortality [67]. Negative impact of chronically increased IL-6 levels on kidney performance during aging was suggested by surveys conducted in the USA and Japan [98]. Age-related alterations in IL-6 signaling have been further linked to insulin resistance which may produce secondary kidney damage, as diabetes mellitus is the leading cause of CKD in industrialized societies [3,90,99]. Hypertension, another meaningful CKD cause, has been linked to inflammation and enhanced IL-6 levels as well [100]. Evaluation of human kidney biopsies along with experimental animal studies suggests that IL-6 potentiates effects of the renin-angiotensin system (RAS) thereby aggravating the hypertensive renal damage [100,101]. Thus, sustained elevation of IL-6 levels may accelerate renal aging via distinct pathophysiological mechanisms.

## 6. Interleukin-6 Signaling in the Aged Kidney

### 6.1. Glomeruli

The glomerular filtration barrier consists of fenestrated capillary endothelial cells and podocytes residing on the joint basement membrane, whereas intraglomerular mesangial cells provide structural and functional support for the glomerular architecture [102,103,104]. Podocytes appear to be the only kidney epithelial cell type expressing IL-6R [105,106]. Therefore, the classic IL-6 signaling mode may play a role in podocyte cell biology. Adult podocytes are terminally differentiated, highly specialized glomerular epithelial cells critical to the intact glomerular filtration barrier [104]. The available data suggests that IL-6 signaling participates in induction of podocyte hypertrophy, which is an adaptive response helping to maintain the intact glomerular filtration barrier by moderate podocyte loss or enlargement of glomeruli size due to increased glomerular workload. Studies in rodents suggest that the ability of podocytes to undergo compensatory hypertrophy reaches its limit during natural aging with ensuing development of age-associated glomerular pathology [107,108]. The transcriptomics signature of aged mouse podocytes is characterized by an inflammatory signaling response with increased expression of TNF, IL-2, and IL-6, suggesting podocyte senescence [109,110]. Inflammatory and senescent podocyte phenotypes have further been reported in mouse models of obesity or hypertension, suggesting that the secondary renal damage may induce premature aging of podocytes [111]. In addition, both classic and trans IL-6 signaling modes have been associated with podocyte injury in diabetes mellitus [112]. It is tempting to speculate that IL-6 contributes to glomerular damage caused by metabolic and cardiovascular disorders, thereby accelerating renal aging.

Senile glomerulosclerosis is associated with moderate mesangial expansion [7,55]. The documented effects of IL-6 on mesangial cells are controversial with some studies reporting pro-inflammatory and proliferative phenotypes, whereas other studies state either no or even inhibitory effects of IL-6 on mesangial cell proliferation and extracellular matrix production [113,114,115]. Experimental studies in cultured human mesangial cells suggested that IL-6 trans-signaling may stimulate recruitment of monocytes via induction of the monocyte chemoattractant protein 1 (MCP-1) [113]. In line with this, a study in mice showed that binding of MCP-1 to the C-C motif chemokine receptor 2 (CCR2) on the surface of non-canonical (patrolling) monocytes leads to their activation, TNF release, and recruitment of neutrophiles within the glomerular microvasculature with ensuing immune-mediated glomerular injury [116]. In contrast, another study found no pathophysiological links between IL-6 signaling in mesangial cells and glomerulonephritis. According to the latter study, IL-6 knockout mice showed largely preserved glomerular architecture and exhibited equally severe phenotypes upon induction of experimental glomerulonephritis as compared to wild-type mice [114]. Moreover, chronic IL-6 infusion failed to produce significant mesangial cell activation or aggravate the experimental mesangial glomerulonephritis in rats [114]. Nevertheless, the specific signaling mode, i.e., classical or trans-signaling, as well as the type of renal pathology, appear to be critical for the effects of the cytokine. It is tempting to speculate that the classic signaling mode exerts adaptive and cytoprotective effects in the glomerulus, whereas the trans-signaling provokes or aggravates glomerulosclerosis. Specific details related to the natural kidney aging remain to be elucidated.

### 6.2. Proximal Tubules

Proximal tubules (PT) reabsorb approximately two-thirds of the filtered NaCl and water, which is an energy-consuming process highly dependent on the appropriate oxygen delivery [117]. Renal plasma flow diminishes with aging causing renal cortical hypoxia, as shown by studies in humans and rodents [118,119,120]. Hypoxia along with oxidative stress are the key drivers of cellular senescence and SASP acquisition by PT cells, which start to secrete IL-6 and other pro-inflammatory cytokines upon these conditions [119,120]. The accumulating number of senescent cells limits the PT functionality, as reflected by the reduced expression of specific PT transport proteins and genes [121]. Apart from the epithelial transport tasks, cortical PTs express Klotho, which has been increasingly recognized as an important renal and systemic anti-aging factor [122]. The a-Klotho protein produced by PT cells is a transmembrane protein acting as a co-receptor for fibroblast growth factor 23 (FGF23) with major impact on renal calcium and phosphate handling and vitamin D metabolism. Binding of FGF23 to the a-Klotho promotes urinary phosphate excretion, thereby preventing hyperphosphatemia and ectopic calcium precipitation [123]. Shedding of the a-Klotho ectodomain by A Disintegrin and Metalloproteinases 10 or 17 (ADAM10 or ADAM17) gives rise to the soluble Klotho variant with various endocrine and paracrine effects mediated by multiple signaling pathways including the Wingless-related integration site (Wnt), insulin/insulin-like growth factor 1 (IGF-1), tumor growth factor b (TGF-β), and nuclear factor kappa-light-chain-enhancer of activated B cells’ (NF-κB) signaling [62,63,122]. The effects of soluble Klotho suppress oxidative stress and inflammation, whereas the transmembrane Klotho form helps to maintain the intact calcium-phosphate homeostasis, thereby preventing hyperphosphatemia and vascular calcification. Chronic inflammation of kidney tissue as it occurs in CKD exerts negative effects on renal Klotho expression [64]. Experimental studies in mice have demonstrated that some pro-inflammatory cytokines such as TNF or the TNF-like weak inducer of apoptosis (TWEAK) suppress renal Klotho expression via the NF-kB signaling [124,125]. As TNF and IL-6 share the downstream NF-kB signaling pathway, chronically enhanced IL-6 levels associated with natural or pathophysiological kidney aging may aggravate the downregulation of Klotho expression, although direct evidence supporting this assumption is pending. Moreover, experimental studies in IL-6 knockout mice suggested that IL-6 stimulates the FGF23 expression via STAT3 [126]. High serum FGF23 levels may exert Klotho-independent effects related to cardiac hypertrophy or liver inflammation [127]. Circulating FGF23 levels tend to increase during aging and are associated with an unfavorable prognosis for cardiovascular, renal, and general health [128,129]. High circulating FGF23 levels may further suppress Klotho expression as part of a negative feedback loop mediated by decrease of active vitamin D levels [130]. In this context, IL-6-dependent stimulation of the FGF23 expression may aggravate the age-dependent suppression of Klotho thereby accelerating the renal and systemic aging.

### 6.3. Distal Nephron and Collecting Ducts

The distal nephron comprises the thick ascending limb (TAL), the distal convoluted tubule (DCT), and the connecting tubule (CNT). Together with the ensuing collecting duct (CD), the segments of the distal nephron enable urinary concentration and fine-tuning of renal electrolyte handling. Effects of pro- and anti-inflammatory cytokines in the distal nephron have been primarily studied in the pathophysiological context of hypertension and inflammation rather than in physiological settings [131]. Only a few experimental studies in cell culture and rodents documented local cytokine signaling in the distal nephron such as inhibition of NaCl reabsorption in TAL by TNF [132]. IL-1 signaling, in turn, has been shown to potentiate NKCC2 activity via paracrine interactions between immune and TAL cells [133]. Tubular effects of IL-6 have not been studied in detail, but the cytokine has been shown to potentiate hypertensive and pro-fibrotic effects of the renin-angiotensin system (RAS) in rodents [134,135]. Sustained elevation of local or systemic IL-6 levels in the aged kidney may, therefore, produce unfavorable effects on tubular function and renal morphology.

Like in the proximal nephron, the cortical segments of the distal nephron express Klotho where it participates in calcium reabsorption [136]. Moreover, evaluation of rodent and human kidneys demonstrated higher Klotho expression in the distal nephron compared to the proximal tubules [137,138]. Apart from the divalent cation reabsorption, Klotho has been implicated in renal potassium handling, which is another principal function of the distal nephron [139]. As mentioned above, little information is available on potential functional interactions between IL-6 and Klotho in the kidney. In non-renal endothelial cells, overexpression of Klotho has been shown to suppress IL-6 expression [140]. Along the same line, exogenous Klotho supplementation improved survival in a COVID-19 mouse model, an effect potentially related to alleviation of the cytokine storm and suppression of IL-6 [141]. Inflammation, in turn, reduces renal Klotho expression, suggesting that IL-6 may be involved herein [64]. Moreover, negative correlations between serum IL-6 and a-Klotho levels have been reported in cardiovascular and renal patients [142,143,144]. Evaluation of aged IL-6 knockout mice and/or older patients receiving IL-6 inhibitors would help to elucidate mutual effects of IL-6 and Klotho during kidney aging.

### 6.4. Renal Vasculature

IL-6 modulates vascular function via effects on endothelial cells mediated by trans-signaling. Excessive circulating or local IL-6 levels may suppress the endothelial nitric oxide synthase (eNOS) activity while strengthening AngII signaling, thereby provoking renal vasoconstriction with ensuing ischemia and oxidative stress of kidney tissue [134,145]. Endothelial cells respond to IL-6 by the release of further inflammatory cytokines such as IL-8, IL-18, and Monocyte Chemoattractant Protein-1 (MCP-1), which enhance vascular permeability and promote inflammation [146]. Elevated serum IL-6 levels have been associated with reduced expression of adiponectin, which is an anti-atherogenic cytokine produced by adipocytes [147]. Consequently, IL-6 has been shown to provoke endothelial lesions in mouse models of atherosclerosis [148]. Both suppression of eNOS and adiponectin have been implicated in vascular disease associated with CKD [86]. Therefore, IL-6 signaling may promote arterial stiffening, remodeling, and sclerosis associated with kidney aging.

### 6.5. Renal Interstitium

Data on direct effects of IL-6 on renal fibroblasts is scarce and in part controversial. IL-6 deficiency had no effects on dynamics of renal fibrosis in a mouse model of obstructive nephropathy produced by unilateral ureteral obstruction (UUO) [149]. In contrast, selective blockade of the IL-6 trans-signaling using a recombinant sgp130 variant (Fc-gp130) has been shown to significantly attenuate UUO-induced kidney fibrosis [150]. Moreover, Fc-gp130 showed anti-fibrotic effects in the ischemia-reperfusion mouse model of kidney injury [150]. The pro-fibrotic effects of IL-6 may combine induction of glomerular damage and tubular atrophy with direct stimulation of fibroblasts, and the latter has been documented in cultured fibroblasts of non-renal origin [151,152]. Thus, IL-6 may contribute to pro-fibrotic changes in kidney aging.

### 6.6. Renal Effects of Interleukin-6 Inhibitors

IL-6 inhibitors have been increasingly recognized as potent anti-inflammatory and immunosuppressive agents and nowadays represent an established strategy to treat various autoimmune and inflammatory diseases including but not limited to rheumatoid arthritis, systemic juvenile idiopathic arthritis, giant cell arteritis, adult-onset Still’s disease, and Castleman’s disease. Several monoclonal antibodies targeting IL-6 or IL-6R have been successfully developed including clazakizumab, olokizumab, siltuximab, sirukumab, tocilizumab, and sarilumab [153]. The most experience in the renal field was collected with tocilizumab, an antibody targeting IL-6R and being the first in class IL-6 inhibiting agent. Tocilizumab has demonstrated a substantial potential for retarding both the T-cell and the antibody-mediated rejection of renal allografts [154,155]. However, other IL-6 inhibiting agents such as olokizumab or olamkicept may have advantages in the context of kidney disease due to alternative mechanisms of action. Olamkicept is a recombinant gp130 modification designed to specifically block IL-6 trans-signaling [156,157]. Since the trans-signaling to a larger extent mediates the pro-inflammatory IL-6 effects, olamkicept is believed to be instrumental in (auto)inflammatory disorders [158]. Olokizumab is an antibody to IL-6 preventing the assembly of the functional hexameric IL-6/IL-6R/gp130 complex by targeting a specific binding site on IL-6 [159]. Due to the specific targeted IL-6 epitope, olokizumab may potentially not only block classic and trans-signaling but also affect trans-presentation, and the latter opportunity needs experimental verification [159].

In general, IL-6 inhibition may bear substantial renoprotective potential in distinct settings of acute or chronic kidney disorders; for review see [153]. However, effects of such agents on natural or pathophysiological kidney aging have received only minor attention so far. An increasing number of patients receiving IL-6-inhibiting therapy for various reasons is expected to provide further renal insights.

## 7. Conclusions

**Figure 2:**
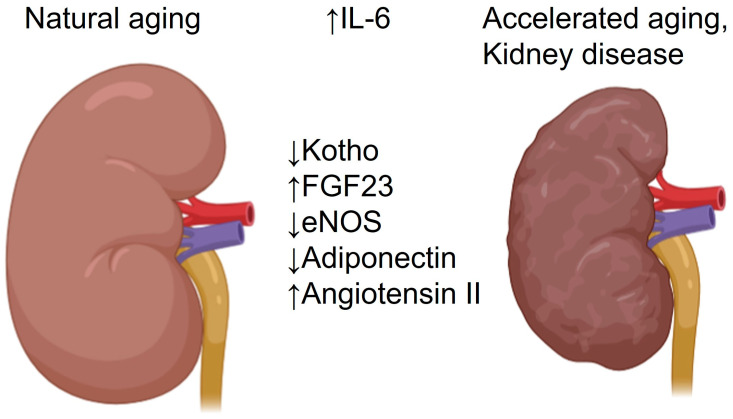
Renal effects of interleukin-6 with potential impact on kidney aging. Elevated systemic or local interleukin-6 (IL-6) concentration leads to suppressed renal production and circulating levels of Klotho, renal activity of the endothelial nitric oxide synthase (eNOS), and circulating levels of adiponectin, while potentiating the renal effects of angiotensin II. These IL-6 effects provoke renal vasoconstriction, hypoxia, and oxidative stress in kidney cells, thereby accelerating natural kidney aging and increasing susceptibility to renal diseases.

Elevated circulating IL-6 levels frequently observed in older people may accelerate kidney aging by several pathophysiological mechanisms including vasoconstriction, epithelial senescence, suppression of Klotho, vascular and tubulo-interstitial inflammation, and pro-fibrotic effects. However, physiological tasks of IL-6 are rather renoprotective, although exact mechanisms and settings defining the adaptive vs. maladaptive cytokine effects remain to be clarified. Potential effects of IL-6 during natural or pathophysiological kidney aging are summarized in Table 2 and Figure 2. Further experimental studies in aged rodent models transgenic for IL-6 signaling components are mandatory for an improved mechanistic understanding of distinct IL-6 effects in the aging kidney. Potential effects of IL-6 on Klotho expression are of particular interest in the context of renal and global aging and require detailed characterization. From the technical point of view, combining modern high-throughput techniques such as spatial transcriptomics with state-of-the-art cellular morphology may advance our understanding of cellular senescence in the context of inflammaging and the role of IL-6 herein.

## Figures and Tables

**Table 1:** Changes of renal functional reserve during natural kidney aging vs. chronic kidney disease progression.

	Natural Kidney Aging	Chronic Kidney Disease
Baseline GFR	Linear decline ~1 mL/min/1.73 m²/year from the age 30 [25]	Stable or non-linear slow decline until critical nephrons loss, then rapid decline [35,36]
Renal functional reserve	Preserved or slightly reduced: ~14–20% up to age 90 [37]	Progressive reduction during disease advancing: 19% → 6.7% (stage 1 → 4) [34]
Clinical implications	Renal compensatory capacity is adequate for stressors	Increased risk of acute kidney disease upon stress

References are given after the respective statements.

**Table 2:** Reported effects of IL-6 in distinct renal cell types and their potential impact on kidney aging.

Cell Type	Adaptive Effects	Maladaptive Effects	References
Podocyte	Hypertrophy → compensation of age-related podocyte loss	Decompensation -> podocyte injury	[112]
Mesangial cell	↓ECM production → ↓glomerulosclerosis	Inflammatory response -> glomerular damage	[113,114,115]
Proximal and distal tubules		SASP, ↑AngII effect, ↓Klotho, ↑FGF23 -> ↓functionality, electrolyte disbalance, vascular calcification, TIN, accelerated aging	[64,119,120,121,124,125,126,136,139]
Vasculature		Vasoconstriction, leakage -> hypoxia, oxidative stress, inflammation, vascular disease	[134,145,147,148]
Interstitium		Direct and indirect pro-fibrotic effects	[150,151,152]

Abbreviations: AngII—angiotensin II, ECM—extracellular matrix, FGF23—fibroblast growth factor 23, SASP—senescence-associated secretory phenotype, TIN—tubule-interstitial nephritis; ↓ indicates reduction, ↑ indicates increase.

## Data Availability

No new data were created or analyzed in this study. Data sharing is not applicable to this article.
